# Patterns of Treatment and Real‐World Outcomes of Patients With Non‐small Cell Lung Cancer With *EGFR* Exon 20 Insertion Mutations Receiving Mobocertinib: The EXTRACT Study

**DOI:** 10.1002/cam4.70369

**Published:** 2025-01-24

**Authors:** Geoffrey Liu, Shi Feng Nyaw, Tony S. K. Mok, Hubert Curcio, Alexis B. Cortot, Tsz Yeung Kam, Renaud Descourt, Yin Kwan Chik, Parneet Cheema, James M. Gwinnutt, Eric N. Churchill, Justin Nyborn, Eileen Curran, Alexandra Savell, Yu Yin, Katie Chong, Yuka Tanaka‐Chambers, Julian Kretz, Jacques Cadranel

**Affiliations:** ^1^ Department of Medical Oncology and Hematology Princess Margaret Cancer Centre Toronto Ontario Canada; ^2^ Department of Clinical Oncology Tuen Mun Hospital Hong Kong China; ^3^ Department of Clinical Oncology The Chinese University of Hong Kong, Prince of Wales Hospital Hong Kong China; ^4^ Oncology Department Centre Francois Baclesse Caen France; ^5^ Centre Hospitalier Universitaire de Lille, CNRS, INSERM Institut Pasteur de Lille, UMR9020 – UMR1277 – Canther – Cancer Heterogeneity, Plasticity and Resistance to Therapies Lille France; ^6^ Department of Clinical Oncology Pamela Youde Nethersole Eastern Hospital Main Block Hong Kong China; ^7^ Oncology Department Hopital Morvan, CHU Brest Brest France; ^8^ Department of Clinical Oncology Queen Elizabeth Hospital Hong Kong China; ^9^ Oncology, William Osler Health System University of Toronto Brampton Ontario Canada; ^10^ Epidemiology and Database Studies IQVIA Reading England UK; ^11^ Global Medical Affairs Oncology Takeda Pharmaceuticals U.S.A., Inc. Lexington Massachusetts USA; ^12^ Global Medical Affairs Oncology, Takeda Development Center Americas, Inc. Lexington Massachusetts USA; ^13^ Global Evidence and Outcome Research Takeda Development Center Americas, Inc. Lexington Massachusetts USA; ^14^ Observational Research Analytics Takeda Development Center Americas, Inc. Lexington Massachusetts USA; ^15^ Clinical Data Management Takeda Development Center Americas, Inc. Lexington Massachusetts USA; ^16^ Statistical and Quantitative Sciences Takeda Development Center Americas, Inc. Lexington Massachusetts USA; ^17^ Medical Affairs Oncology – EUCan Takeda Pharmaceuticals International AG Zurich Switzerland; ^18^ Pulmonology and Thoracic Oncology Department APHP Hôpital Tenon and Sorbonne Université Paris France

**Keywords:** EGFR exon 20 insertion, mobocertinib, non‐small cell lung cancer, real‐world outcomes

## Abstract

**Background:**

Real‐world data regarding patients with non‐small cell lung cancer (NSCLC) with *EGFR* exon 20 insertion (*ex20ins*) mutations receiving mobocertinib are limited. This study describes these patients' characteristics and outcomes.

**Methods:**

A chart review was conducted across three countries (Canada, France, and Hong Kong), abstracting data from eligible patients (NCT05207423). The inclusion criteria were: ≥ 18 years old; diagnosis of stage IIIB‐IV NSCLC with *EGFR ex20ins* between January 1, 2017 and November 30, 2021; received mobocertinib. Data on demographics, clinical parameters, treatment patterns, mobocertinib exposure, real‐world outcomes, and adverse events (AEs) were collected. Results are also reported by Asian/Non‐Asian races.

**Results:**

Overall, 105 patients were enrolled (median [IQR] age at initial diagnosis: 64.0 years [56, 71]; women: 62.9%). The most common first‐line of therapy (LoT) was chemotherapy; the most common second LoT was EGFR tyrosine kinase inhibitors. Most patients received mobocertinib during LoT two and three (74.3%); the maximum dose was 160 mg/day for 67.6% of the cohort (mean [SD] daily dose: 130.6 mg [36.68]). The median real‐world progression‐free survival (PFS) on mobocertinib was 4.76 months (95% CI: 3.98, 6.21). The overall response rate and disease control rate were 20.0% and 48.6%, respectively (median duration of response: 8.34 months [95% CI: 3.61, 9.49]). The median overall survival (OS) was 26.28 months (95% CI: 20.21, 36.44). Asian patients had numerically superior PFS and OS compared with non‐Asian patients. Regarding safety analysis, 73 patients (69.5%) experienced any AE. The most common AE was diarrhea (any grade) (52 patients; 49.5%).

**Conclusions:**

These data illustrate the real‐world effectiveness of mobocertinib.

## 1 Introduction

Non‐small cell lung cancer (NSCLC) accounts for approximately 85% of all lung cancers [1]. Approximately 10%–40% of NSCLC tumors have mutations in the epidermal growth factor receptor (*EGFR*) gene [2, 3]. The most common mutations are exon 19 deletions and exon 21 and exon 18 substitutions [1]. *EGFR* exon 20 insertion (*ex20ins*) mutations are estimated to comprise 0.1%–4.0% of all NSCLC cases and 4%–12% of patients with *EGFR* mutations [4, 5, 6]. *EGFR* mutations appear to be more common in women, Asian patients, never tobacco users, older patients, and those with adenocarcinoma histology [5, 6, 7]. In contrast, other reports suggest there is no variation in *EGFR ex20in* prevalence by race [4, 8]. Asian patients with NSCLC may also have improved outcomes compared with other race groups [9, 10, 11].

In contrast to common *EGFR* mutations, tumors with *ex20ins* mutations are less sensitive to EGFR tyrosine kinase inhibitors (TKIs) developed to target common *EGFR* mutations (e.g., gefitinib, erlotinib, afatinib, or osimertinib). Therefore, patients with NSCLC and *EGFR ex20ins* mutations typically have worse outcomes compared to patients with common *EGFR* mutations (exon 19 deletions and L858R point mutations on exon 21) [12]. A recent systematic review reported that the overall response rate (ORR) for patients with *ex20ins* mutations treated with TKIs ranged from 0%‐20% (7 studies; 194 patients), compared with 27.4%–84% for patients with common *EGFR* mutations treated with TKIs (5 studies; 1193 patients) [6]. Likewise, median progression‐free survival (PFS) ranged from 1.4–3.0 months for patients with *ex20ins* mutations treated with TKIs (8 studies; 183 patients), compared with 8.5–15.2 months for patients with common *EGFR* mutations treated with TKIs (3 studies; 501 patients) [6].

Drugs have been developed with the aim of improving outcomes for patients with NSCLC and *EGFR ex20ins* mutations, including amivantamab and mobocertinib [13]. A study of 114 platinum‐pretreated patients (PPP cohort) with metastatic NSCLC and *EGFR ex20ins* treated with 160 mg/day of mobocertinib (combining a dose escalation study, an expansion study in seven molecularly and histologically defined expansion cohorts, and an extension study) reported an independent review committee (IRC)‐assessed confirmed objective response rate of 28% and a median IRC‐assessed PFS of 7.3 months (95% confidence interval [CI]: 5.5, 9.2) [14]. Patients who received mobocertinib also maintained their quality of life over time and had improvements in self‐reported lung cancer symptoms [15]. In terms of safety, a pooled analysis of three cohorts of patients (*N* = 257) with NSCLC and *EGFR ex20ins* mutations receiving mobocertinib (160 mg/day) reported that the most common treatment‐related adverse events (AEs) were diarrhea (235 patients; 91%), followed by nausea (102 patients; 40%) and rash (94 patients; 37%) [16]. In October 2023, it was announced that mobocertinib will be withdrawn from the market as the EXCLAIM‐2 confirmatory study did not meet the primary endpoint [17].

Real‐world data from patients with NSCLC and *EGFR ex20ins* mutations receiving mobocertinib are limited. The current analysis aimed to generate further data on treatment patterns and outcomes in patients receiving mobocertinib in a real‐world setting. The objectives of this analysis were to describe (i) the demographics and clinical characteristics, (ii) patterns of care, (iii) real‐world outcomes, and (iv) safety outcomes of patients with NSCLC and *EGFR ex20ins* mutations. Furthermore, given the potential variation in outcomes by race in NSCLC and the higher prevalence of *EGFR ex20ins* mutations in Asian patients, this study also investigated outcomes based on Asian/Non‐Asian race.

## 2 Material And Methods

### 2.1 Study Design

The EXTRACT study (Exon 20 Insertion Retrospective Analysis of Patient CharT Data; NCT05207423) was a retrospective chart review of patients from three countries: Canada, France, and Hong Kong. In total, 29 sites extracted data from the charts of patients who were eligible for the study. Data were abstracted from medical records from the date of initial diagnosis until the data abstraction date. Data collection started on October 03, 2022 in Hong Kong, October 11, 2022 in France, and November 21, 2022 in Canada; the database was locked on May 23, 2023.

### 2.2 Patients

Patients were included in the EXTRACT study if they were:

- Aged ≥ 18 years old;
- Had histologically/cytologically confirmed diagnosis of locally advanced or metastatic (stage IIIB to IV) NSCLC with *EGFR ex20ins* mutations (based on the evaluation by the treating center) between January 01, 2017 and November 30, 2021;
- Were followed up at their site between January 01, 2017 and November 30, 2021 for their advanced NSCLC, irrespective of their current survival status;
- One of the following:
  ○ Site waiver for informed consent requirement obtained;
  ○ Patient consent was obtained for the collection of their medical data.

Furthermore, in addition to the inclusion criteria, patients who met the following exclusion criterion were not included in the study:

- Patients whose investigator had access to fewer than two registered visits for his/her advanced NSCLC between January 01, 2017 and November 30, 2021.

Patients were included in the EXTRACT analysis if they received mobocertinib in at least one line of therapy (LoT).

Three patients were diagnosed outside the inclusion window of January 01, 2017 and November 30, 2021; however, these patients were retained in the analysis population (diagnosis dates: June 04, 2015; December 01, 2016; January 14, 2022).

### 2.3 Data Collection and Outcome Definitions

Demographic data were abstracted from medical charts, including age at initial diagnosis, sex, race, tobacco use, occupation, and comorbidities present at advanced diagnosis. No data on race were available for patients from France, as these data were not collected as per local regulations.

NSCLC characteristics, year of initial and advanced diagnosis, histological type of NSCLC, anatomic stage at advanced diagnosis, Eastern Cooperative Oncology Group (ECOG) status at advanced diagnosis [18], and location of first metastasis were abstracted from medical records. The frequency of brain metastasis was also recorded. Patients were classified into three groups of insertion mutations depending on the position of their insertion in *exon 20*: near loop (positions 767–772); far loop (positions 773–775); and other positions [19].

Anticancer therapies by LoT were also abstracted, including start and stop dates as well as investigator assessments of response and progression. Therapies are only summarized in this report for LoT 5 due to the small sample size for LoT ≥ 6. The following regimen categories were used to categorize the anticancer therapies:

- Chemotherapy: carboplatin (platinum‐based), cisplatin (platinum‐based), docetaxel, etoposide, gemcitabine, paclitaxel, paclitaxel protein‐bound, pemetrexed, topotecan, and vinorelbine.
- EGFR TKIs: afatinib, erlotinib, gefitinib, osimertinib, dacomitinib, mobocertinib, zipalertinib, and sunvozertinib.
- Monoclonal antibodies: cetuximab, ramucirumab, bevacizumab, bevacizumab‐awwb, necitumumab, ado‐trastuzumab emtansine, and amivantamab.
- Immuno‐oncologic (IO) agents: atezolizumab, durvalumab, ipilimumab, nivolumab, and pembrolizumab.

Regarding mobocertinib exposure, the time from advanced diagnosis to treatment start, duration of exposure, average daily dose, and maximum daily dose are reported.

Several real‐world outcomes were calculated based on abstracted data using the following definitions:

- Real‐world progression‐free survival (rwPFS): evaluated from the initiation of the mobocertinib LoT to real‐world progressive disease or death from any cause. Patients with no documented evidence of progression or death within a LoT were censored the day before the next LoT or date of last contact.
- Real‐world overall response rate (rwORR): proportion of patients who achieved complete (rwCR) or partial response (rwPR) during the mobocertinib LoT.
- Real‐world confirmed overall response rate (rwCORR): proportion of patients who achieved rwCR or rwPR during the mobocertinib LoT and response persisted for ≥ 4 weeks.
- Real‐world disease control rate (rwDCR): proportion of patients who achieved rwCR, rwPR, or real‐world stable disease during the mobocertinib LoT.
- Real‐world duration of response (rwDOR): evaluated from the date of first rwCR or rwPR during the mobocertinib LoT to the date of progressive disease or death. The same censoring definition used for rwPFS was also used in the analysis of rwDOR.
- Overall survival (OS): evaluated from the date of advanced disease diagnosis to the date of death. Patients for whom no date of death was identified were censored at the date of last contact.
- Real‐world time to treatment discontinuation (rwTTD): evaluated from treatment initiation to treatment discontinuation for any reason; time during temporary interruptions was included in the calculation of rwTTD. Treatment discontinuation was defined as the date of the last drug administered as part of the mobocertinib LoT, or date of death. For patients' last LoT, patients without a recorded date of death were censored at the date of last contact.

Reported AEs were coded into System Organ Classes and preferred terms using MedDRA version 25.0. The numbers and proportions of patients experiencing AEs and serious adverse events (SAEs) are reported, along with incidence rates and 95% CI. The numbers and proportions of patients experiencing gastrointestinal AEs, including diarrhea and nausea/vomiting, are also reported.

### 2.4 Subgroup Analyses

Analyses are presented for all patients in the study, and a pre‐specified stratification based on race category (Asian; non‐Asian) is also included. Patients whose race was recorded as Asian were included in the “Asian” category; all other patients were included in the “Non‐Asian” category. Note that race could not be collected in France, and as such, all patients from France were considered “race not reported” and included in the “Non‐Asian” category.

### 2.5 Statistical Analysis

Descriptive statistics were used to summarize the demographic and clinical characteristics of the cohort. Kaplan–Meier methodology was used to summarize time‐to‐event outcomes and reported as median and 95% CI. Exact binomial 95% CIs were calculated around binary endpoints (rwORR, rwCORR, rwDCR). No imputation was carried out for missing data, other than missing dates; where days were missing, the day was replaced by the middle day of the month. SAS Software version 9.4 was used for all statistical analyses. The Sankey plot was generated using R 4.2.0.

## 3 Results

### 3.1 Demographic Characteristics and Medical History

In total, 105 patients were included in the analysis, including 39 men (37.1%) and 66 women (62.9%). The median age at initial NSCLC diagnosis was 64.0 years (interquartile range [IQR]: 56, 71), and four patients (3.8%) were current tobacco users, 39 patients (37.1%) were former tobacco users, and 58 patients (55.2%) were never users of tobacco (tobacco use was unknown for four patients). Of 77 patients with non‐missing race data, 41 patients (53.2%) were Asian, eight patients (10.4%) were White, and 28 patients (36.4%) were reported as having no race data available (Table 1). Of the total sample, 51 patients (48.6%) came from France, 19 patients (18.1%) came from Canada, and 35 patients (33.3%) came from Hong Kong.

**TABLE 1:** Demographic characteristics.

	All patients	Asian	Non‐Asian^a^
*N*	105	41	64
Sex, *N* (%)			
Male	39 (37.1%)	16 (39.0%)	23 (35.9%)
Female	66 (62.9%)	25 (61.0%)	41 (64.1%)
Age at initial NSCLC diagnosis (years), median (IQR)	64.0 (56, 71)	62.0 (56, 70)	65.0 (57, 72)
Race, *N* (%)			
American Indian or Alaska Native	0	0	0
Asian	41 (53.2%)	41 (100%)	0
Black or African American	0	0	0
Native Hawaiian or Other Pacific Islander	0	0	0
White	8 (10.4%)	0	8 (22.2%)
Multiple races reported	0	0	0
Race not reported	28 (36.4%)	0	28 (77.8%)
Missing	28	0	28
Tobacco use, *N* (%)			
Current	4 (3.8%)	0	4 (6.3%)
Former	39 (37.1%)	12 (29.3%)	27 (42.2%)
Never	58 (55.2%)	25 (61.0%)	33 (51.6%)
Unknown	4 (3.8%)	4 (9.8%)	0
Comorbidities at the time of NSCLC diagnosis, *N* (%)^b^			
COPD	2 (1.9%)	0	2 (3.1%)
Asthma	6 (5.7%)	1 (2.4%)	5 (7.8%)
Liver impairment	1 (1.0%)	1 (2.4%)	0
Renal failure, neuropathy	2 (1.9%)	1 (2.4%)	1 (1.6%)
Cardiovascular disease	11 (10.5%)	3 (7.3%)	8 (12.5%)
Cerebrovascular disease	4 (3.8%)	0	4 (6.3%)
Hypertension (medically treated)	24 (22.9%)	9 (22.0%)	15 (23.4%)
Hypercholesterolemia	4 (3.8%)	2 (4.9%)	2 (3.1%)
Diabetes	14 (13.3%)	8 (19.5%)	6 (9.4%)
Chronic viral infection (Hepatitis B, Hepatitis C, HIV)	2 (1.9%)	1 (2.4%)	1 (1.6%)
Osteoarthritis	2 (1.9%)	1 (2.4%)	1 (1.6%)
Rheumatoid arthritis	2 (1.9%)	1 (2.4%)	1 (1.6%)
Other malignancies (excluding NSCLC)	13 (12.4%)	4 (9.8%)	9 (14.1%)
Other	27 (25.7%)	10 (24.4%)	17 (26.6%)

Abbreviations: COPD = chronic obstructive pulmonary disorder; HIV = human immunodeficiency virus; IQR = interquartile range; *N* = number; NSCLC = non‐small cell lung cancer.

^a^ Includes patients of unknown race.

^b^ Comorbidities are not mutually exclusive.

The demographic characteristics of the Asian patients (*n* = 41) and non‐Asian (*n* = 64) patients were similar, other than tobacco use, whereby more non‐Asian patients were former tobacco users, whereas more Asian patients were never tobacco users (Table 1).

### 3.2 NSCLC at Advanced Diagnosis

Three patients (2.9%) had stage IIIB disease at time of advanced diagnosis, 99 patients (96.1%) had stage IV, and one patient (1.0%) was recorded as “other” (stage IIIC) (two patients had missing data). The histological type of NSCLC was primarily adenocarcinoma (102 patients [97.1%]), and the majority of patients received their advanced diagnosis in 2019 or 2020 (N [%]: < 2017: two patients [1.9%]; 2017: six patients [5.7%]; 2018: seven patients [6.7%]; 2019: 34 patients [32.4%]; 2020: 36 patients [34.3%]; 2021: 19 patients [18.1%]; 2022: one patient [1.0%]). The majority of patients had ECOG performance status (PS) of 0 (30 patients [30.3%]) or 1 (56 patients [56.6%]) at advanced diagnosis, with the remaining patients having more impaired function (ECOG PS 2 or 3: 13 patients [13.1%]; missing PS: six patients).

The most common sites of metastasis at the time of diagnosis of advanced disease were bone (35 patients [33.3%]), followed by brain/central nervous system (19 patients [18.1%]), and then the contralateral lung (11 patients [10.5%]).

Furthermore, 21 patients (20.0%) had their first metastatic site recorded as “other,” which mainly comprised patients who had metastases at more than one location (see Table S1 for Asian/Non‐Asian patients' NSCLC characteristics).

Per the inclusion criteria, all patients had an *EGFR ex20ins* detected; 62 patients (59.0%) had near loop mutations, 15 patients (14.3%) had far loop mutations, and nine patients (8.6%) had mutations in other positions (in the remaining 19 patients [18.1%], mutation positions were not identified). Seventeen Asian patients (41.5%) and 45 non‐Asian patients (70.3%) had near loop mutations.

### 3.3 Anticancer Treatments by Regimen Category

**FIGURE 1:**
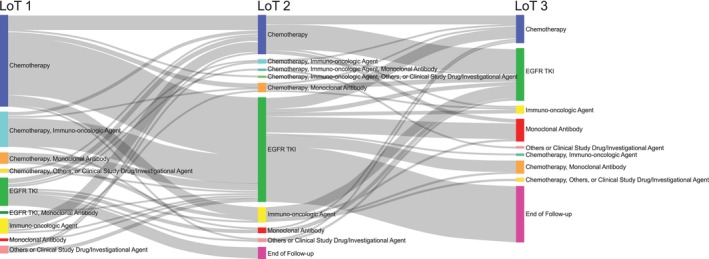
Sankey plot of the first three LoTs, by regimen category. The Sankey plot illustrates the proportions of patients receiving combinations of each regimen category at each LoT, from LoT 1 to LoT 3, as well as the treatment patterns. Each combination of regimen categories is represented by a unique color, and the nodes (bars) are labeled. The drugs that comprise each regimen category are listed in the Methods section. The LoT is listed at the top of each column and also indicated in square brackets in the node labels. The size of each node in each LoT column is proportional to the number of patients receiving that combination of regimen categories during that LoT. The gray paths between nodes are proportional to the number of patients who changed from one combination of regimen categories to another between LoTs and represent the patient's journey between different regimens. The Sankey plot represents treatment patterns for systemic anticancer therapies. Radiotherapy and surgery are not included. EGFR TKI = epidermal growth factor receptor tyrosine kinase inhibitor; IO agents = immuno‐oncologic agents; LoT = line of therapy.

The most common treatment received by patients in LoT 1 was chemotherapy (76 patients [72.4%]) (Table 2 and Figure 1). After LoT 1, the proportion of patients who received chemotherapy plateaued between 30.3% (LoT 2) and 41.5% (LoT 4). Regarding race stratification, 78.1% of the non‐Asian patients received chemotherapy in LoT 1 compared with 63.4% of the Asian patients, whereas in later lines the proportions were similar, although there was variation due to the small sample size. Immunotherapy agents were most commonly used in LoT 1 (25.7%), with the proportion below 12.1% in subsequent LoTs.

**TABLE 2:** Anticancer treatments by line of therapy.

	LoT 1, *N* (%)	LoT 2, *N* (%)	LoT 3, *N* (%)	LoT 4, *N* (%)	LoT 5, *N* (%)
Number of patients receiving each LoT					
Overall	105	99	69	41	17
Asian	41	39	25	13	7
Non‐Asian	64	60	44	28	10
Chemotherapy, *N* (%)					
Overall^a^	76 (72.4%)	30 (30.3%)	24 (34.8%)	17 (41.5%)	7 (41.2%)
Asian^b^	26 (63.4%)	14 (35.9%)	8 (32.0%)	7 (53.8%)	1 (14.3%)
Non‐Asian^c^	50 (78.1%)	16 (26.7%)	16 (36.4%)	10 (35.7%)	6 (60.0%)
EGFR TKI, *N* (%)					
Overall^a^	16 (15.2%)	56 (56.6%)	28 (40.6%)	14 (34.1%)	5 (29.4%)
Asian^b^	11 (26.8%)	20 (51.3%)	11 (44.0%)	4 (30.8%)	3 (42.9%)
Non‐Asian^c^	5 (7.8%)	36 (60.0%)	17 (38.6%)	10 (35.7%)	2 (20.0%)
Mobocertinib, *N* (%)					
Overall^a^	9 (8.6%)	53 (53.5%)	26 (37.7%)	12 (29.3%)	4 (23.5%)
Asian^b^	4 (9.8%)	20 (51.3%)	10 (40.0%)	4 (30.8%)	2 (28.6%)
Non‐Asian^c^	5 (7.8%)	33 (55.0%)	16 (36.4%)	8 (28.6%)	2 (20.0%)
Amivantamab, *N* (%)					
Overall^a^	1 (1.0%)	3 (3.0%)	12 (17.4%)	6 (14.6%)	5 (29.4%)
Asian^b^	1 (2.4%)	2 (5.1%)	4 (16.0%)	0	3 (42.9%)
Non‐Asian^c^	0	1 (1.7%)	8 (18.2%)	6 (21.4%)	2 (20.0%)
IO Agents, *N* (%)					
Overall^a^	27 (25.7%)	12 (12.1%)	5 (7.2%)	2 (4.9%)	0
Asian^b^	8 (19.5%)	4 (10.3%)	1 (4.0%)	2 (15.4%)	0
Non‐Asian^c^	19 (29.7%)	8 (13.3%)	4 (9.1%)	0	0
Monoclonal antibodies, *N* (%)					
Overall^a^	8 (7.6%)	9 (9.1%)	19 (27.5%)	10 (24.4%)	6 (35.3%)
Asian^b^	3 (7.3%)	4 (10.3%)	4 (16.0%)	1 (7.7%)	3 (42.9%)
Non‐Asian^c^	5 (7.8%)	5 (8.3%)	15 (34.1%)	9 (32.1%)	3 (30.0%)

*Note:* Treatments are not mutually exclusive. The Definition of treatment categories is included in Methods section.

Abbreviations: EGFR TKI = epidermal growth factor receptor tyrosine kinase inhibitor; IO = Immuno‐oncologic; LoT = line of therapy; *N* = number.

^a^ Denominator is the number of patients at each LoT (provided in the “N total” row)—for example, 76 of 105 patients (72.4%) received chemotherapy at LoT 1.

^b^ Denominator for the Asian treatment row is the number of Asian patients within the LoT (provided in the “N total” row)—for example, 26 of 41 Asian patients (63.4%) at LoT 1 received chemotherapy.

^c^ Denominator for the non‐Asian treatment row is the number of non‐Asian patients within the LoT (provided in the “N total” row); for example, 50 of 64 non‐Asian patients (78.1%) at LoT 1 received chemotherapy.

Only 15.2% of patients received an EGFR TKI in LoT 1, whereas EGFR TKIs were the most common treatment in LoT 2 (56 of 99 patients [56.6%]). After LoT 2, the proportion of patients who received an EGFR TKI decreased, with 29.4% receiving an EGFR TKI in LoT 5. Of 41 Asian patients, 26.8% received an EGFR TKI in LoT 1, compared with 7.8% of the non‐Asian patients; similar proportions of Asian and non‐Asian patients received EGFR TKIs in LoT 2 and LoT 3.

One patient (1.0%) received amivantamab in LoT 1, three patients (3.0%) in LoT 2, 12 patients (17.4%) in LoT 3, six patients (14.6%) in LoT 4, and five patients (29.4%) in LoT 5.

### 3.4 Mobocertinib Exposure

Patients primarily received mobocertinib in either the second or third LoT (78 patients [74.3%]). One patient received mobocertinib in two LoTs. There was a median of 12.5 months (IQR: 7.8, 19.8) from advanced NSCLC diagnosis to mobocertinib initiation, and patients were exposed to mobocertinib for a median of 4.2 months (IQR: 2.0, 9.3). The majority of patients received a maximum daily dose of 160 mg (71 patients [67.6%]); however, a number of patients never received this dose during the study (maximum daily dose 120 mg: 16 patients [15.2%]; 80 mg: eight patients [7.6%]; 40 mg: six patients [5.7%]) (Table 3). The mean (SD) daily dose was 130.6 mg/day (36.68). Twenty‐five patients (23.8%) received mobocertinib followed by amivantamab (Table S2), and five patients (4.8%) received amivantamab followed by mobocertinib.

**TABLE 3:** Exposure to mobocertinib.

	All patients	Asian	Non‐Asian
*N*	105	41	64
LoT patients received mobocertinib, *N* (%)			
1	9 (8.6%)	4 (9.8%)	5 (7.8%)
2	53 (50.5%)	20 (48.8%)	33 (51.6%)
3	26 (24.8%)	10 (24.4%)	16 (25.0%)
2 and/or 3^a^	78 (74.3%)	30 (73.2%)	48 (75.0%)
4	12 (11.4%)	4 (9.8%)	8 (12.5%)
5	4 (3.8%)	2 (4.9%)	2 (3.1%)
6	2 (1.9%)	1 (2.4%)	1 (1.6%)
Time from advanced NCSLC diagnosis to mobocertinib start [months], median (IQR)	12.5 (7.8, 19.8)	14.9 (6.5, 25.3)	10.8 (7.9, 16.6)
Duration of exposure [months], median (IQR)	4.2 (2.0, 9.3)	5.8 (3.3, 11.7)	3.5 (1.4, 7.0)
Average daily dose [mg/day], median (IQR)	160.0 (120.0, 160.0)	129.4 (120.0, 160.0)	160.0 (114.1, 160.0)
Maximum daily dose administered, *N* (%)			
160 mg	71 (67.6%)	22 (53.7%)	49 (76.6%)
120 mg	16 (15.2%)	11 (26.8%)	5 (7.8%)
80 mg	8 (7.6%)	5 (12.2%)	3 (4.7%)
40 mg	6 (5.7%)	1 (2.4%)	5 (7.8%)
Missing	4	2	2

Abbreviations: IQR = interquartile range; LoT = line of therapy; mg = milligrams; *N* = number.

^a^ One (non‐Asian) patient received mobocertinib in LoT 2 and LoT 3 – this row combines LoT 2 and LoT 3 and includes the patient only once.

The proportion of Asian patients and non‐Asian patients who received mobocertinib during LoT 2 and/or LoT 3 was similar. However, the time from advanced NSCLC diagnosis to mobocertinib start was shorter for non‐Asian patients. Furthermore, non‐Asian patients received mobocertinib for a shorter period of time at higher doses compared with Asian patients (Table 3).

### 3.5 Real‐World Outcomes

The real‐world outcomes of the patients are presented in Table 4, for all LoTs where mobocertinib was received combined and stratified by the LoT in which patients received mobocertinib (up to LoT 5).

**TABLE 4:** Real‐world outcomes, all LoTs combined and by LoT.

	All LoTs combined	LoT 1	LoT 2	LoT 3	LoT 4	LoT 5
rwPFS^a^, median (95% CI) [*N*]						
Overall	4.76 (3.98, 6.21) [105]	3.45 (0.20, 14.72) [9]	4.76 (3.68, 6.87) [53]	5.13 (3.12, 10.02) [25]	3.98 (0.76, 8.74) [12]	3.94 (1.25, NA) [4]
Asian	6.47 (4.76, 11.50) [41]	9.08 (1.74, NA) [4]	6.41 (3.68, 16.00) [20]	10.64 (1.12, NA) [10]	8.74 (6.47, NA) [4]	3.94 (NA, NA) [2]
Non‐Asian	3.98 (2.53, 5.03) [64]	3.45 (0.20, NA) [5]	3.84 (2.53, 6.80) [33]	4.47 (1.05, 7.69) [15]	1.81 (0.49, 4.63) [8]	3.96 (1.25, NA) [2]
rwORR, % (95% CI^b^) [*n*/*N*]						
Overall	20% (12.8, 28.9) [21/105]	11.1% (0.3, 48.2) [1/9]	18.9% (9.4, 32.0) [10/53]	24.0% (9.4, 45.1) [6/25]	25.0% (5.5, 57.2) [3/12]	0% (0.0, 60.2) [0/4]
Asian	19.5% (8.8, 34.9) [8/41]	0% (0.0, 60.2) [0/4]	20.0% (5.7, 43.7) [4/20]	20.0% (2.5, 55.6) [2/10]	50.0% (6.8, 93.2) [2/4]	0% (0.0, 84.2) [0/2]
Non‐Asian	20.3% (11.3, 32.2) [13/64]	20.0% (0.5, 71.6) [1/5]	18.2% (7.0, 35.5) [6/33]	26.7% (7.8, 55.1) [4/15]	12.5% (0.3, 52.7) [1/8]	0% (0.0, 84.2) [0/2]
rwCORR, % (95% CI^b^) [*n*/*N*]						
Overall	17.1% (10.5, 25.7) [18/105]	11.1% (0.3, 48.2) [1/9]	15.1% (6.7, 27.6) [8/53]	20.0% (6.8, 40.7) [5/25]	25.0% (5.5, 57.2) [3/12]	0% (0.0, 60.2) [0/4]
Asian	14.6% (5.6, 29.2) [6/41]	0% (0.0, 60.2) [0/4]	10.0% (1.2, 31.7) [2/20]	20.0% (2.5, 55.6) [2/10]	50.0% (6.8, 93.2) [2/4]	0% (0.0, 84.2) [0/2]
Non‐Asian	18.8% (10.1, 30.5) [12/64]	20.0% (0.5, 71.6) [1/5]	18.2% (7.0, 35.5) [6/33]	20.0% (4.3, 48.1) [3/15]	12.5% (0.3, 52.7) [1/8]	0% (0.0, 84.2) [0/2]
rwDCR, % (95% CI^b^) [*n*/*N*]						
Overall	48.6% (38.7, 58.5) [51/105]	44.4% (13.7, 78.8) [4/9]	47.2% (33.3, 61.4) [25/53]	56.0% (34.9, 75.6) [14/25]	41.7% (15.2, 72.3) [5/12]	50.0% (6.8, 93.2) [2/4]
Asian	46.3% (30.7, 62.6) [19/41]	25.0% (0.6, 80.6) [1/4]	35.0% (15.4, 59.2) [7/20]	70.0% (34.8, 93.3) [7/10]	50.0% (6.8, 93.2) [2/4]	100% (15.8, 100) [2/2]
Non‐Asian	50.0% (37.2, 62.8) [32/64]	60.0% (14.7, 94.7) [3/5]	54.5% (36.4, 71.9) [18/33]	46.7% (21.3, 73.4) [7/15]	37.5% (8.5, 75.5) [3/8]	0% (0.0, 84.2) [0/2]
rwDOR^a^, median (95% CI) [*N*]						
Overall	8.34 (3.61, 9.49) [21]	2.86 (NA, NA) [1]	9.23 (1.18, NA) [10]	7.13 (2.00, NA) [6]	7.95 (7.72, NA) [3]	—
Asian	7.72 (2.10, 9.49) [8]	—	NA (2.10, NA) [4]	7.13 (4.76, NA) [2]	7.84 (7.72, NA) [2]	—
Non‐Asian	9.23 (2.86, 15.05) [13]	2.86 (NA, NA) [1]	9.28 (1.18, NA) [6]	9.33 (2.00, NA) [4]	8.34 (NA, NA) [1]	—
rwTTD^a^, median (95% CI) [*N*]						
Overall	4.50 (3.45, 6.08) [105]	4.50 (0.20, 8.97) [9]	4.21 (3.32, 6.83) [53]	5.16 (1.81, 8.11) [25]	3.12 (0.82, 10.25) [12]	5.24 (1.25, NA) [4]
Asian	6.83 (4.93, 11.37) [41]	4.81 (1.84, NA) [4]	8.02 (3.25, 16.00) [20]	6.97 (0.10, 12.02) [10]	10.25 (5.19, NA) [4]	9.61 (3.84, NA) [2]
Non‐Asian	3.48 (2.40, 4.93) [64]	3.45 (0.20, NA) [5]	4.04 (2.43, 5.62) [33]	3.45 (0.89, 7.20) [15]	1.86 (0.20, 3.75) [8]	3.94 (1.25, NA) [2]
OS^a^, median (95% CI) [*N*]						
Overall	26.28 (20.21, 36.44) [105]	—	—	—	—	—
Asian	37.65 (25.82, 50.04) [41]	—	—	—	—	—
Non‐Asian	20.44 (16.92, 34.10) [64]	—	—	—	—	—
Patients with brain metastasis, *N* (%)						
Overall	48 (45.7%)	—	—	—	—	—
Asian	16 (39.0%)	—	—	—	—	—
Non‐Asian	32 (50.0%)	—	—	—	—	—

*Note:* Table displays real‐world outcomes for all treatment lines combined and stratified by line of LoT in which patients received mobocertinib; one patient received mobocertinib in LoT 2 and LoT 3, only their first exposure to mobocertinib (LoT 2) is considered in this table. Furthermore, two patients received mobocertinib in LoT 6. These patients are only included in the ‘All LoTs combined’ column.

Abbreviations: CI = confidence interval; LoT = line of therapy; OS = overall survival; rwCORR = real‐world complete overall response rate; rwDCR = real‐world disease control rate; rwDOR = real‐world duration of response; rwORR = real‐world overall response rate; rwPFS = real‐world progression‐free survival; rwTTD = real‐world time to treatment discontinuation; *n* = number of patients with response; *N* = number of patients in the analysis; NA = not applicable.

^a^ Presented in months.

^b^ Exact binomial CI.

Regarding rwPFS, 84 patients (80.0%) had an event (progression: 68 patients [64.8%]; death: 16 patients [15.2%]) and 21 patients (20.0%) were censored. The rwPFS while on mobocertinib was 4.76 months (95% CI: 3.98, 6.21) for all LoTs combined. Patients who received mobocertinib in LoT 2 and LoT 3 appeared to have a numerically longer median rwPFS compared with patients who received mobocertinib in other LoTs. The median rwPFS for Asian and non‐Asian patients were 6.47 months (95% CI: 4.76, 11.50) and 3.98 months (95% CI: 2.53, 5.03), respectively.

Median PFS was broken out by insertion type and was collected for the mobocertinib cohort; however, this was not broken out by line of therapy. For patients with near loop insertions (positions 767–772), the median PFS was 4.47 months (95% CI: 3.32, 5.62; *n* = 62), and patients with far loop insertions (positions 773–775) had a numerically longer median PFS of 5.16 months (95% CI: 1.74, 6.87; *n* = 15).

The rwORR, rwCORR, and rwDCR were 20% (95% CI: 12.8, 28.9), 17.1% (95% CI: 10.5, 25.7), and 48.6% (95% CI: 38.7, 58.5), respectively, for all LoTs combined. The response rates were similar across the LoTs. Asian and non‐Asian patients had similar response rates, although with some variation across the LoTs.

The median rwDOR in the 21 patients who responded to mobocertinib and had available data was 8.34 months (95% CI: 3.61, 9.49). The median rwDOR for patients who responded to mobocertinib by LoT was 9.23 months (95% CI: 1.18, not applicable [NA]), 7.13 months (95% CI: 2.00, NA), and 7.95 months (95% CI: 7.72, NA) at LoT 2, 3, and 4, respectively. The median rwDOR for Asian and non‐Asian patients were 7.72 months (95% CI: 2.10, 9.49) and 9.23 months (95% CI: 2.86, 15.05), respectively.

The median rwTTD for all LoTs combined in which mobocertinib was received was 4.50 months (95% CI: 3.45, 6.08). The median rwTTD for Asian and non‐Asian patients were 6.83 months (95% CI: 4.93, 11.37) and 3.48 months (95% CI: 2.40, 4.93), respectively.

Seventy‐two patients (68.6%) died during the study period, and the OS was 26.28 months (95% CI: 20.21, 36.44). The median OS for Asian and non‐Asian patients were 37.65 months (95% CI: 25.82, 50.04) and 20.44 months (95% CI: 16.92, 34.10), respectively.

### 3.6 Safety Analysis

In total, 73 patients (69.5%) experienced an AE during mobocertinib treatment. The most common System Organ Class was gastrointestinal disorders, wherein 60 patients (57.1%) experienced a gastrointestinal AE; 52 patients (49.5%) experienced diarrhea; and 22 patients (21.0%) experienced nausea and/or vomiting. All but one of these gastrointestinal AEs were reported to be related to mobocertinib treatment (Table 5). In total, 14 patients (19.2%) had treatment withdrawn due to an AE, and 30 patients (41.1%) had their dose reduced due to an AE.

**TABLE 5:** Safety events during mobocertinib treatment.

	All patients	Asian	Non‐Asian
*N*	105	41	64
Patients with any AE, *N* (%)	73 (69.5%)	27 (65.9%)	46 (71.9%)
Patients with any SAE, *N* (%)	15 (14.3%)	2 (4.9%)	13 (20.3%)
Patients with any AE causing a dose or drug modification^a^, *N* (%)	50 (47.6%)	15 (36.6%)	35 (54.7%)
Patients with any AE leading to death, *N* (%)	4 (3.8%)	0	4 (6.3%)
GI AEs, *N* (%)			
Any GI AE	60 (57.1%)	24 (58.5%)	36 (56.3%)
Any GI AE related to mobocertinib	59 (56.2%)	23 (56.1%)	36 (56.3%)
Diarrhea AEs, *N* (%)			
Any diarrhea AE	52 (49.5%)	20 (48.8%)	32 (50.0%)
Any diarrhea AE related to mobocertinib	51 (48.6%)	19 (46.3%)	32 (50.0%)
Nausea/vomiting AEs, *N* (%)			
Any nausea/vomiting AE	22 (21.0%)	9 (22.0%)	13 (20.3%)
Any nausea/vomiting AE related to mobocertinib	22 (21.0%)	9 (22.0%)	13 (20.3%)

Abbreviations: AE = adverse event; GI = gastrointestinal; *N* = number.

^a^ Dose or drug modification = dose reduced, dose rate reduced, drug interrupted, drug withdrawn, dose delayed, drug infusion interrupted, all drugs withdrawn, dose increased.

Regarding SAEs, 15 patients (14.3%) experienced an SAE during mobocertinib treatment, including six patients (5.7%) who experienced gastrointestinal‐related SAEs, most commonly diarrhea (three patients [2.9%]).

A similar proportion of Asian patients and non‐Asian patients experienced any AEs (Asian: 27 patients [65.9%]; non‐Asian: 46 patients [71.9%]). Two Asian patients (4.9%) and 13 non‐Asian patients (20.3%) experienced SAEs. The proportion of patients who experienced gastrointestinal events was similar between Asian and non‐Asian patients (Table 5).

## 4 Discussion

This analysis summarized the real‐world treatment management and clinical outcomes of 105 patients with NSCLC with *EGFR ex20ins* mutations who were part of the EXTRACT study and who received mobocertinib. Two‐thirds of these patients were female, and the median age was 64 years. Approximately half of the patients came from France, a third from Hong Kong, and the remaining patients from Canada. The majority of patients were diagnosed with advanced disease in 2019 and 2020; these patients primarily had adenocarcinomas and only small limitations in functional ability at diagnosis. These characteristics were similar to other recent publications, indicating generalizability [4, 20, 21, 22].

Patients primarily received chemotherapy during the first LoT and EGFR TKIs (including mobocertinib) during the second and third LoTs. Patients typically received mobocertinib approximately 1 year after advanced diagnosis on average. While two‐thirds of patients received a maximum daily dose of 160 mg, there were many patients who received lower doses. Patients typically received IO agents in earlier LoTs and amivantamab and other monoclonal antibodies in later LoTs, although this was before the publication of the PAPILLON clinical trial demonstrating efficacy of amivantamab + chemotherapy compared with chemotherapy monotherapy as first‐line treatment [23].

Regarding real‐world outcomes, the median rwPFS was 4.76 months for patients taking mobocertinib. This is lower than the PFS reported by Zhou et al. (7.3 months for the PPP cohort) and was longer than the median rwPFS reported from studies of patients with NSCLC and *EGFR ex20ins* mutations receiving other EGFR TKIs (1.4–3.0 months; eight studies [183 patients]) [6]. Likewise, a recent meta‐analysis by Kwon et al. of eight studies of patients receiving EGFR TKIs reported a pooled PFS of 3.0 months (95% CI: 2.0, 3.8) [24]. Regarding confirmed response rates, patients in EXTRACT had slightly lower rwCORR when compared with the cORR from independent review committee assessments from Zhou et al. (EXTRACT: 17.1%; PPP: 28%) [14]. The rwORR from EXTRACT was at the high end of the range of rwORRs reported from previous analyses of patients with NSCLC and *EGFR ex20ins* receiving other TKIs (0%–20%) summarized by Burnett et al. [6], and higher than the pooled estimate from Kwon et al. (6.8% [95% CI: 3.3, 13.5]) [24]. Regarding OS, patients from EXTRACT and patients included in the PPP cohort of Zhou et al.'s analysis had similar median survival (EXTRACT: 26.28 months; PPP: 24.0 months) [14], higher than patients receiving previous EGFR TKIs (Burnett et al. [6]: 4.8–19 months; six studies [177 patients]; Kwon et al. [24]: 16.4 months [95% CI: 11.6, 19.7]).

In summary, the real‐world effectiveness of mobocertinib as demonstrated in EXTRACT is in line with what was previously observed in clinical trials [14]. Historically, real‐world effectiveness of drugs is typically lower than efficacy seen in clinical trials due to the highly controlled nature of clinical trials [25]. Furthermore, patients in this real‐world analysis typically received lower doses than patients in Zhou et al. (160 vs. 130.6 mg), supporting a narrow therapeutic window for mobocertinib. However, compared with previous analyses of patients receiving other EGFR TKIs, patients in the EXTRACT study had superior outcomes. A separate adjusted analysis reported patients receiving mobocertinib had superior outcomes compared with platinum chemotherapy (CORR odds ratio = 3.75 [95% CI: 2.05, 6.89]; PFS hazard ratio = 0.57 [95% CI: 0.39, 0.90]) [26].

Fewer patients in EXTRACT experienced AEs compared with Zhou et al. (any AE: EXTRACT: 69.5%; PPP: 100%) [14]. Diarrhea was the most commonly reported AE in EXTRACT and Zhou et al., as well as other reported analyses. However, whereas 91% of patients in the PPP cohort of Zhou et al. reported diarrhea, only 49.5% of the EXTRACT cohort reported diarrhea (48.6% related to mobocertinib); a similar rate of diarrhea related to mobocertinib was reported in a previous real‐world evidence study by Kian et al. (52%), albeit with a small sample size (*N* = 16) [27]. Potential explanations include the retrospective nature of the cohort, and patients in EXTRACT received lower doses of mobocertinib on average compared with Zhou et al., as previous analyses have demonstrated a relationship between increased dose and greater risk of AEs [28]. Furthermore, real‐world management of AEs and requirements for dose reductions are different in a real‐world setting compared with clinical trials.

Within EXTRACT, Asian patients tended to have better outcomes compared with non‐Asian patients, including longer rwPFS, rwTTD, and OS, but response rates (rwORR, rwCORR, rwDCR) and rwDOR were similar (potentially due to Asian patients with stable disease having longer PFS compared with non‐Asian patients). However, as the Asian patients predominantly came from Hong Kong, it is unclear if these differences are due to race or country‐level differences in management. Furthermore, as race data in France are not routinely collected, any Asian patients from France will have been misclassified into the non‐Asian group (the number of Asian patients in France is expected to be low). However, as this misclassification will bias the results toward the null, any differences in outcomes between Asian and non‐Asian patients observed within this study may be an underestimation of the true difference.

This study has a number of strengths. The international setting of the study allows greater generalization of the results. The comprehensive data on treatment patterns allow an in‐depth understanding of the way patients with NSCLC and *EGFR ex20ins* are managed. However, there are several limitations to this study. Many patients received other targeted therapies prior to starting mobocertinib, which could influence results. There were some missing data, potentially biasing results. Standard of care practice evolves over time and is variable from country to country. This limits the collection of standardized and complete information on key data such as brain imaging and AE management. Lastly, as response and progression were assessed by investigators at each site, there may be some variation in the assessments, which will lead to greater variability in the data and could also bias the results.

## 5 Conclusion

The EXTRACT study enrolled a large and representative cohort of patients with NSCLC and *EGFR ex20ins* mutations who received mobocertinib, reporting on treatment management patterns, real‐world outcomes, and safety. The results indicate the clinical effectiveness and relative tolerability of mobocertinib when used in a real‐world setting. Unfortunately, with the recent report of the negative confirmatory study (EXCLAIM‐2), mobocertinib is being withdrawn globally.

## Author Contributions

**Geoffrey Liu:** data curation (equal), investigation (equal), resources (equal), writing – review and editing (equal). **Shi Feng Nyaw:** data curation (equal), investigation (equal), resources (equal), writing – review and editing (equal). **Tony S. K. Mok:** data curation (equal), investigation (equal), resources (equal), writing – review and editing (equal). **Hubert Curcio:** data curation (equal), investigation (equal), resources (equal), writing – review and editing (equal). **Alexis B. Cortot:** data curation (equal), investigation (equal), resources (equal), writing – review and editing (equal). **Tsz Yeung Kam:** data curation (equal), investigation (equal), resources (equal), writing – review and editing (equal). **Renaud Descourt:** data curation (equal), resources (equal), writing – review and editing (equal). **Yin Kwan Chik:** data curation (equal), investigation (equal), resources (equal), writing – original draft (equal), writing – review and editing (equal). **Parneet Cheema:** data curation (equal), investigation (equal), resources (equal), writing – review and editing (equal). **James M. Gwinnutt:** formal analysis (equal), writing – original draft (equal), writing – review and editing (equal). **Eric N. Churchill:** conceptualization (equal), formal analysis (equal), writing – review and editing (equal). **Justin Nyborn:** conceptualization (equal), formal analysis (equal), writing – review and editing (equal). **Eileen Curran:** conceptualization (equal), formal analysis (equal), writing – review and editing (equal). **Alexandra Savell:** formal analysis (equal), writing – review and editing (equal). **Yu Yin:** conceptualization (equal), formal analysis (equal), writing – review and editing (equal). **Katie Chong:** data curation (equal), writing – review and editing (equal). **Yuka Tanaka‐Chambers:** data curation (equal), formal analysis (equal), writing – review and editing (equal). **Julian Kretz:** conceptualization (equal), formal analysis (equal), writing – review and editing (equal). **Jacques Cadranel:** conceptualization (equal), formal analysis (equal), investigation (equal), resources (equal), writing – review and editing (equal).

## Ethics Statement

This study (A Chart Review Study of Adults With Advanced NSCLC [EXTRACT]; NCT05207423) was approved by applicable country institutional review boards or ethics committees, and the study was conducted in accordance with the Declaration of Helsinki and Guidelines for Good Pharmacoepidemiology Practice. Due to the retrospective nature of the study, waivers for the requirement to obtain informed consent were obtained for Canada and Hong Kong (waivers were not required in France as per local regulations). Two sites in Hong Kong did not receive waivers and were required to obtain informed consent.

## Conflicts of Interest

Geoffrey Liu has received honoraria from and served on advisory boards for Takeda, Amgen, AstraZeneca, Roche, Novartis, Merck, Pfizer, Jazz Pharmaceuticals, Bristol Myers Squibb, Anheart, Eli Lilly, EMD Serono, Bayer, and Jansen and has received research grants from Takeda, AstraZeneca, Amgen, Boehringer Ingelheim, Bayer, EMD Serono, and Pfizer. Shi Feng Nyaw reports no conflict of interest. Tony S. K. Mok has acted in a consulting or advisory role for AbbVie, ACEA Pharma, Alpha Biopharma, Amgen, Amoy Diagnostics, AstraZeneca, BeiGene, Boehringer Ingelheim, Bristol Myers Squibb, Blueprint Medicines, CStone Pharmaceuticals, Daiichi Sankyo, Eisai, Fishawack Facilitate, GeneDecode, Gritstone Oncology Inc., Guardant Health, Hengrui Therapeutics, Ignyta Inc., IQVIA, Incyte, Janssen, Lilly, Loxo Oncology, Lunit USA, Merck Serono, Merck Sharp & Dohme, Mirati Therapeutics, MORE Health, Novartis, OrigiMed, Pfizer, Puma Biotechnology, Roche, Sanofi‐Aventis R&D, Takeda, Virtus Medical Group, Yuhan Corp., SFJ Pharmaceuticals, Curio Science, Inivata, Berry Oncology, G1 Therapeutics Inc., Qiming Development (HK) Ltd., Gilead Sciences, Vertex Pharmaceuticals, Covidien LP, Elevation Oncology, and C4 Therapeutics; has been an invited speaker for ACEA Pharma, Alpha Biopharma, Amgen, Amoy Diagnostics, AstraZeneca, BeiGene, Boehringer Ingelheim, Bristol Myers Squibb, Daiichi Sankyo, Fishawack Facilitate, GeneDecode, InMed Medical Communication, Janssen, Eli Lilly, Lunit USA, MD Health, Medscape/WebMD, Merck Serono, Merck Sharp & Dohme, Novartis, OrigiMed, PeerVoice, Physicians' Education Resource, P. Permanyer SL, Pfizer, PrIME Oncology, Research to Practice, Roche, Sanofi‐Aventis R&D, Takeda, Touch Medical Media, Daz Group, Lucence Health, Merck Pharmaceuticals HK Ltd., Shanghai BeBirds Translation & Consulting Co., Llangylhul Network Technology Co., and Taiho; has served as a member of the board of directors for Lunit USA, AstraZeneca PLC, Hutchison Chi‐Med, Act Genomics‐Sanomics Group, and Aurora; reports stock ownership for Hutchison Chi‐Med, Act Genomics‐Sanomics Group, and Aurora; and has received funding from Clovis Oncology and Xcovery. Hubert Curcio reports honoraria from and advisory board participation for AstraZeneca, Bristol Myers Squibb, and Takeda and registration fees and accommodation from Sandoz and GlaxoSmithKline. Alexis B. Cortot has been an invited speaker for Pfizer, Amgen, Takeda, Novartis, Roche, AstraZeneca, Merck Sharp & Dohme, Janssen, Bristol Myers Squibb, and Sanofi; acted in a consulting or advisory role for Novartis, AbbVie, Roche, Exeliom, Pfizer, Janssen, Amgen, Takeda, AstraZeneca, and Merck Sharp & Dohme; received research funding of institution from Exeliom; received support for attending meetings from Roche, Merck Sharp & Dohme, Novartis, Pfizer, AstraZeneca, Amgen, and Bristol Myers Squibb; and participated on DMSB for InhaTarget and Merck. Tsz Yeung Kam reports no conflict of interest. Renaud Descourt has served on the advisory boards for AstraZeneca, Bristol Myers Squibb, Merck Sharp & Dohme, Pfizer, Sanofi, and Takeda. Yin Kwan Chik reports no conflict of interest. Parneet Cheema has served on the advisory boards for Amgen, AstraZeneca, Bristol Myers Squibb, Bayer, Novartis, Roche, Pfizer, Janssen, Merck, Sanofi, and GlaxoSmithKline, and has received honoraria from GlaxoSmithKline, Janssen, Sanofi, Merck, and Amgen. James M. Gwinnutt is an employee of IQVIA. Eric N. Churchill is an employee of Takeda. Justin Nyborn is an employee of Takeda. Eileen Curran reports former employment with Takeda. Alexandra Savell is an employee of Takeda. Yu Yin is an employee of Takeda. Katie Chong is an employee of Takeda. Yuka Tanaka‐Chambers reports former employment with Takeda. Julian Kretz reports former employment with Takeda. Jacques Cadranel has served on advisory boards for Amgen, AstraZeneca, Boehringer Ingelheim, Bristol Myers Squibb, Janssen, Merck Sharp & Dohme, Novartis, Pfizer, Sanofi, and Takeda.

## Third‐Party Submission

Editorial and submission support were provided by Peloton Advantage, LLC, an OPEN Health company, and funded by Takeda Development Center Americas Inc. The authors have authorized a third party to submit on their behalf and have approved the manuscript draft submitted by the third party.

## Supporting information


Table S1.

Table S2.

## Data Availability

The datasets, including the redacted study protocol, redacted statistical analysis plan, and individual participant data supporting the results reported in this article, will be made available within 3 months from the initial request to researchers who provide a methodologically sound proposal. The data will be provided after its de‐identification, in compliance with applicable privacy laws, data protection, and requirements for consent and anonymization.
