# Supplementary material for: Patterns of Treatment and Real‐World Outcomes of Patients With Non‐small Cell Lung Cancer With EGFR Exon 20 Insertion Mutations Receiving Mobocertinib: The EXTRACT Study
Source: Cancer Med. 2025 Jan 24;14(3):e70369. doi: 10.1002/cam4.70369 (PMC11761427; doi:10.1002/cam4.70369)
Supplement: Supplementary file 1 — Table S1. [file CAM4-14-e70369-s002.docx]

Table A.1 – NSCLC Characteristics

|  | **All patients** | **Asian** | **Non-Asian** |
| --- | --- | --- | --- |
| **N** | 105 | 41 | 64 |
| **Calendar year of advanced diagnosis**, N (%) |  |  |  |
| <2017 | 2 (1.9%) | 1 (2.4%) | 1 (1.6%) |
| 2017 | 6 (5.7%) | 3 (7.3%) | 3 (4.7%) |
| 2018 | 7 (6.7%) | 4 (9.8%) | 3 (4.7%) |
| 2019 | 34 (32.4%) | 11 (26.8%) | 23 (35.9%) |
| 2020 | 36 (34.3%) | 12 (29.3%) | 24 (37.5%) |
| 2021 | 19 (18.1%) | 10 (24.4%) | 9 (14.1%) |
| 2022 | 1 (1.0%) | 0 | 1 (1.6%) |
| Missing | 0 | 0 | 0 |
| **Anatomic stage at advanced diagnosis**, N (%) |  |  |  |
| IIIB | 3 (2.9%) | 2 (5.0%) | 1 (1.6%) |
| IV | 99 (96.1%) | 38 (95.0%) | 61 (96.8%) |
| Other, IIIC | 1 (1.0%) | 0 | 1 (1.6%) |
| Missing | 2 | 1 | 1 |
| **Histological type of NSCLC**, N (%) |  |  |  |
| Adenocarcinoma, NOS | 101 (96.2%) | 39 (95.1%) | 62 (96.9%) |
| Adenocarcinoma with mixed subtypes | 1 (1.0%) | 1 (2.4%) | 0 |
| Carcinoma, undifferentiated, NOS | 1 (1.0%) | 1 (2.4%) | 0 |
| Mixed cell adenocarcinoma | 1 (1.0%) | 0 | 1 (1.6%) |
| Squamous cell carcinoma, large cell, non-keratinizing, NOS | 1 (1.0%) | 0 | 1 (1.6%) |
| **ECOG performance status at advanced diagnosis**, N (%) |  |  |  |
| 0 | 30 (30.3%) | 11 (28.2%) | 19 (31.7%) |
| 1 | 56 (56.6%) | 22 (56.4%) | 34 (56.7%) |
| 2 | 11 (11.1%) | 6 (15.4%) | 5 (8.3%) |
| 3 | 2 (2.0%) | 0 | 2 (3.3%) |
| 4 | 0 | 0 | 0 |
| Missing | 6 | 2 | 4 |
| **Location of first metastatic disease**, N(%) |  |  |  |
| Brain, CNS | 19 (18.1%) | 10 (24.4%) | 9 (14.1%) |
| Bone | 35 (33.3%) | 14 (34.1%) | 21 (32.8%) |
| Lung (other than initial location, contralateral lung) | 11 (10.5%) | 2 (4.9%) | 9 (14.1%) |
| Pleura | 9 (8.6%) | 7 (17.1%) | 2 (3.1%) |
| Lymph nodes (mediastinal, cervical, infraclavicular, supraclavicular) | 5 (4.8%) | 2 (4.9%) | 3 (4.7%) |
| Liver | 3 (2.9%) | 1 (2.4%) | 2 (3.1%) |
| Adrenal glands | 2 (1.9%) | 0 | 2 (3.1%) |
| Other | 21 (20.0%) | 5 (12.2%) | 16 (25.0%) |
| Missing | 0 | 0 | 0 |
| CNS = central nervous system, NOS = not otherwise specified, NSCLC = Non-small cell lung cancer | | | |
