# Supplementary material for: Patterns of Treatment and Real‐World Outcomes of Patients With Non‐small Cell Lung Cancer With EGFR Exon 20 Insertion Mutations Receiving Mobocertinib: The EXTRACT Study
Source: Cancer Med. 2025 Jan 24;14(3):e70369. doi: 10.1002/cam4.70369 (PMC11761427; doi:10.1002/cam4.70369)
Supplement: Supplementary file 2 — Table S2. [file CAM4-14-e70369-s001.docx]

| Table B.1 – Real-world outcomes during amivantamab treatment in the cohort of patients who received amivantamab post mobocertinib | | | |
| --- | --- | --- | --- |
| RW outcome | **All patients** | **Asian** | **Non-Asian** |
| **rwPFS^†^** |  |  |  |
| N | 25 | 9 | 16 |
| Median (95% CI) | 4.70 (1.51, 5.95) | 2.04 (0.85, NA) | 5.65 (1.41, 7.92) |
| **rwORR** |  |  |  |
| N | 25 | 9 | 16 |
| % (95% CI) [n] | 24.0% (9.4, 45.1) [6] | 11.1% (0.3, 48.2) [1] | 31.3% (11.0, 58.7) [5] |
| **rwCORR** |  |  |  |
| N | 25 | 9 | 16 |
| % (95% CI) [n] | 20.0% (6.8, 40.7) [5] | 11.1% (0.3, 48.2) [1] | 25.0% (7.3, 52.4) [4] |
| **rwDCR** |  |  |  |
| N | 25 | 9 | 16 |
| % (95% CI) [n] | 48.0% (27.8, 68.7) [12] | 33.3% (7.5, 70.1) [3] | 56.3% (29.9, 80.2) [9] |
| **rwDOR^†^** |  |  |  |
| N | 6 | 1 | 5 |
| Median (95% CI) | 4.27 (1.64, NA) | 3.78 (NA, NA) | 4.99 (1.64, NA) |
| **rwTTD†** |  |  |  |
| N | 25 | 9 | 16 |
| Median (95% CI) | 3.52 (1.22, 5.19) | 2.00 (0.03, 4.44) | 4.14 (0.79, 7.69) |
| **OS†** |  |  |  |
| N | 25 | 9 | 16 |
| Median (95% CI) | 39.00 (26.28, NA) | 54.11 (26.02, NA) | 37.09 (19.65, NA) |
| † presented in months  CI = confidence interval, OS = overall survival, n = number of patients with response, N = number of patients in the analysis, NA = not applicable, RW = real-world; rwCORR = real-world complete overall response rate, rwDCR = real-world disease control rate, rwDOR = real-world duration of response, rwORR = real-world overall response rate, rwPFS = real-world progression free survival, rwTTD = real-world time to treatment discontinuation | | | |
